# Weanling pigs consume more feed if hybrid rye replaces corn in diets, but average daily gain and fecal scores are not impacted by hybrid rye

**DOI:** 10.1093/tas/txad022

**Published:** 2023-02-19

**Authors:** Molly L McGhee, Jessica P Acosta, Hans H Stein

**Affiliations:** Department of Animal Sciences, University of Illinois, Urbana, IL 61801, USA; Division of Nutritional Sciences, University of Illinois, Urbana, IL 61801, USA; Department of Animal Sciences, University of Illinois, Urbana, IL 61801, USA; Division of Nutritional Sciences, University of Illinois, Urbana, IL 61801, USA

**Keywords:** cereal grains, corn, growth performance, hybrid rye, weanling pigs

## Abstract

An experiment was conducted to test the hypothesis that growth performance and health status of pigs will not be reduced if hybrid rye is included in diets at the expense of corn during the initial 5 wk post-weaning. A total of 128 weanling pigs (5.6 ± 0.5 kg) were randomly allotted to 32 pens and 4 dietary treatments. Pigs were fed experimental diets for 35 d in three phases with days 1 to 7 being phase 1, days 8 to 21 being phase 2, and days 22 to 35 being phase 3. Within each phase, a control diet primarily based on corn and soybean meal was formulated, and three additional diets were formulated by including 8.0, 16.0, or 24.0% (phase 1), 16.0, 32.0, or 48.0% (phase 2), and 20.0, 40.0, or 60.3% (phase 3) hybrid rye in the diet at the expense of corn. Pig weights were recorded at the start and conclusion of each phase, fecal scores were visually assessed every other day on a pen basis, and blood samples were obtained from 1 pig per pen on days 21 and 35. Results indicated that average daily gain (ADG) in phase 1 increased (linear, *P* < 0.05) as the inclusion of hybrid rye increased, but no other differences in ADG were observed. Average daily feed intake linearly increased in phase 1, phase 3, and overall (*P* < 0.05) as hybrid rye inclusion increased in the diets, and gain:feed was negatively impacted by the inclusion of hybrid rye in the diet (phase 1, linear, *P* < 0.05; phases 2, 3, and overall, quadratic, *P* < 0.05). No differences in average fecal scores or diarrhea incidence were observed. On days 21 and 35, blood urea N increased (linear, *P* < 0.05) as hybrid rye increased in the diets; and on day 21, serum total protein also increased (linear, *P* < 0.05) with increasing hybrid rye inclusion in the diet. Mean blood hemoglobin concentration on day 35 increased and then decreased as hybrid rye inclusion increased (quadratic, *P* < 0.05). On day 21, interleukin (IL) 2 and IL 10 decreased and then increased (quadratic, *P* < 0.05) as hybrid rye inclusion increased. On day 35, IL 8 and IL 12 increased and then decreased (quadratic, *P* < 0.05) and interferon-gamma decreased and then increased (quadratic, *P* < 0.01) as hybrid rye inclusion increased. In conclusion, the ADG of pigs was not different among treatments, but at the highest hybrid rye inclusion level, pigs consumed more feed than if corn was fed and gain:feed was reduced with increasing hybrid rye in diets. Differences in blood serum cytokines indicate the immune system was affected differently when hybrid rye instead of corn was fed.

## INTRODUCTION

In a previous experiment, no differences in final body weights were observed when hybrid rye was fed to nursery pigs at inclusion rates of up to 12, 21, and 60% in phases 1 (days 1 to 7 post-weaning), 2 (days 8 to 21 post-weaning), and 3 (days 22 to 35 post-weaning), respectively (McGhee and Stein, 2021). Average daily feed intake (ADFI) was not affected until phase 3, at which point feed intake quadratically increased as hybrid rye in the diet increased. Based on these results, it was hypothesized that greater inclusion rates of hybrid rye may be used in phases 1 and 2 without negatively impacting growth performance. Hybrid rye contains more dietary fiber than corn, and in growing pigs, the fermentability of dietary fiber from hybrid rye was greater than the fermentability of dietary fiber from corn (McGhee and Stein, 2020). Fermentation of arabinoxylans and fructooligosaccharides, which are abundant in hybrid rye, increases the proportion of beneficial bacteria (bifidobacteria and lactobacilli) in the hindgut of monogastric animals (Bach Knudsen and Lærke, 2010; Bouhnik et al., 2007; Hughes et al., 2007; Schokker et al., 2018). Consumption of rye also increases the quantity of butyrate and the proportion of butyrate in total volatile fatty acids in the colon (Bach Knudsen and Lærke, 2010; Bach Knudsen et al., 1991, 2005). Butyrate has anti-inflammatory effects in the colonal digesta and anticarcinogenic effects in the colon, as well as the potential to improve gut barrier integrity (Hamer et al., 2008; Vogt et al., 2014). In the previous experiment, a reduction in the incidence of diarrhea was observed in the first week after weaning when small amounts of hybrid rye were included in the diet, and differences in blood characteristics were observed among treatments. It is however, not known if greater concentrations of hybrid rye will have additional health benefits, and it is not known whether or not newly weaned pigs are able to consume sufficient energy to maintain growth performance if hybrid rye replaces a greater proportion of corn than in the previous experiment. Therefore, an experiment was conducted to test the hypothesis that feeding diets with greater inclusion rates of hybrid rye at the expense of corn in phase 1 and phase 2 diets for weanling pigs may beneficially influence health parameters without reducing growth performance.

## MATERIALS AND METHODS

The experiment was conducted at the Swine Research Center at the University of Illinois following a protocol that was approved by the Institutional Animal Care and Use Committee at the University of Illinois Urbana-Champaign (Urbana, IL, USA).

### Animals, Housing, and Experimental Design

A total of 128 pigs that were the offspring of Line 359 boars and Camborough sows (Pig Improvement Company, Henderson, TN, USA) with an initial body weight of 5.6 ± 0.5 kg were weaned at 19 to 21 d of age and allotted to four treatment groups. Thirty-two pens were used with four pigs per pen and eight replicate pens per treatment. Weaning weight was used as the blocking factor, sex was balanced among pens, and treatments were randomly allotted to the pens. Pigs were housed in an environmentally controlled nursery barn with fully slatted floors, a nipple waterer, and a stainless-steel feeder. Water and feed were available on an *ad libitum* basis.

From d 14 post-farrowing and until weaning, pigs were offered a commercial creep feed according to normal farm practices. This diet was based on corn and soybean meal as well as animal proteins and did not contain hybrid rye. Pigs were fed experimental diets for 35 d after weaning and a three-phase feeding program was used. Phase 1 diets were fed for 7 d post-weaning, phase 2 diets were fed from days 8 to 21, and phase 3 diets were fed from days 22 to 35 post-weaning. A total of 12 diets were formulated (Table 1), and all diets were fed in dry meal form. Cereal grains were ground using a hammer mill (model: MM4; Schutte Buffalo, NY, USA). In each phase, a control diet primarily based on corn and soybean meal and three diets in which increasing quantities of hybrid rye replaced corn were formulated. In phase 1, the inclusion rate of hybrid rye in each diet was 0, 8.0, 16.0, or 24.0%. In phase 2, the inclusion rate of hybrid rye was 0, 16.0, 32.0, or 48.0%, whereas the inclusion rate of hybrid rye in phase 3 diets was 0, 20.0, 40.0, or 60.3%. All diets were formulated to meet or exceed the estimated requirements for standardized ileal digestible amino acids, vitamins, and minerals for pigs in each of the three phases (NRC, 2012). However, diets were not formulated to be isocaloric or isonitrogenous; therefore, as hybrid rye inclusion rate increased within each phase, the calculated concentrations of total dietary fiber and crude protein increased, whereas the concentration of metabolizable energy decreased.

**Table 1.** Ingredient composition of diets with increasing replacement of corn with hybrid rye

	Phase 1^1^				Phase 2^1^				Phase 3^1^			
Ingredient, %	0%	20%	40%	60%	0%	30%	60%	90%	0%	33%	66%	100%
Ground corn	39.96	31.98	23.97	15.97	53.40	37.41	21.43	5.45	60.27	40.29	20.33	-
Ground hybrid rye	-	8.00	16.00	24.00	-	16.00	32.00	48.00	-	20.00	40.00	60.34
Whey, dried	25.00	25.00	25.00	25.00	12.00	12.00	12.00	12.00	-	-	-	-
Soybean meal	20.00	20.00	20.00	20.00	24.50	24.50	24.50	24.50	35.00	35.00	35.00	35.00
Fish meal	8.00	8.00	8.00	8.00	6.00	6.00	6.00	6.00	-	-	-	-
Blood plasma	3.50	3.50	3.50	3.50	-	-	-	-	-	-	-	-
Soybean oil	2.00	2.00	2.00	2.00	2.00	2.00	2.00	2.00	2.00	2.00	2.00	2.00
Ground limestone	0.76	0.74	0.74	0.74	0.87	0.90	0.94	0.99	0.86	0.91	0.97	1.01
Dicalcium phosphate	-	-	-	-	0.25	0.19	0.12	0.05	1.05	0.97	0.87	0.80
L-lysine HCl, 78%	0.21	0.20	0.20	0.20	0.34	0.34	0.33	0.32	0.25	0.24	0.23	0.22
DL-methionine, 98%	0.09	0.10	0.11	0.11	0.10	0.12	0.13	0.14	0.07	0.08	0.09	0.11
L-threonine, 98%	0.03	0.03	0.03	0.03	0.09	0.09	0.10	0.10	0.05	0.06	0.06	0.07
Salt	0.30	0.30	0.30	0.30	0.30	0.30	0.30	0.30	0.30	0.30	0.30	0.30
Vitamin-mineral premix^2^	0.15	0.15	0.15	0.15	0.15	0.15	0.15	0.15	0.15	0.15	0.15	0.15

^1^The percentages indicate the amount of corn that was replaced with hybrid rye in the diets.

^2^The vitamin-micromineral premix provided the following quantities of vitamins and microminerals per kg of complete diet: Vitamin A as retinyl acetate, 11,136 IU; vitamin D_3_ as cholecalciferol, 2,208 IU; vitamin E as DL-alpha tocopheryl acetate, 66 IU; vitamin K as menadione dimethylprimidinol bisulfite, 1.42 mg; thiamin as thiamine mononitrate, 0.24 mg; riboflavin, 6.59 mg; pyridoxine as pyridoxine hydrochloride,0.24 mg; vitamin B_12_, 0.03 mg; D-pantothenic acid as D-calcium pantothenate, 23.5 mg; niacin, 44.1 mg; folic acid, 1.59 mg; biotin, 0.44 mg; Cu, 20 mg as copper sulfate and copper chloride; Fe, 126 mg as ferrous sulfate; I, 1.26 mg as ethylenediamine dihydriodide; Mn, 60.2 mg as manganese sulfate; Se, 0.3 mg as sodium selenite and selenium yeast; and Zn, 125.1 mg as zinc sulfate.

Individual pig weights were recorded at the beginning of the experiment and at the conclusion of each phase. Feed additions were recorded daily, and feed left in the feeders at the conclusion of each phase was weighed to calculate feed disappearance. Fecal scores were assessed visually for each pen as a whole every other day by two independent observers using a score from 1 to 5 (1 = normal feces; 2 = moist feces; 3 = mild diarrhea; 4 = severe diarrhea; and 5 = watery diarrhea).

### Blood Sample Collection and Analyses

Two 3-mL blood samples were collected from the jugular vein from one pig per pen on the last day of phase 2, as well as on the last day of phase 3. An equal number of barrows and gilts were sampled within treatment, and the same pigs were sampled at each time point. Of the two samples of blood obtained per pig, one was collected in a vacutainer containing ethylenediamine-tetraacetic acid as an anticoagulant, immediately placed on ice, and analyzed for complete blood cell count on a multiparameter automated hematology analyzer (CELL-DYN 3700, Abbott Laboratories, Abbott Park, IL, USA). The second sample of blood was collected in a serum vacutainer containing spray-dried silica as a clot activator. Samples were allowed to clot and then centrifuged at 4,000 × *g* for 13 min at room temperature. Serum was removed from centrifuged tubes and stored at −20°C until analysis. Total protein, blood urea N, and albumin were analyzed using a Beckman Coulter Clinical Chemistry AU analyzer (Beckman Coulter Inc., Brea, CA, USA). The concentration of immunoglobulin G (IgG) in serum samples was determined by enzyme-linked immunosorbent assay following the manufacturer’s instructions (Bethyl Laboratories, Inc., Montgomery, TX, USA). Concentrations of interleukin (IL)-1α, IL-1β, IL-1 receptor antagonist (IL-1RA), IL-2, IL-4, IL-6, IL-8, IL-10, IL-12, IL-18, interferon-gamma (IFN-γ), and tumor necrosis factor-α in serum samples were measured via a porcine-specific multiplex immunoassay kit (MilliporeSigma, Burlington, MA, USA) and read with a Luminex MagPix instrument (Luminex Corporation, Austin, TX, USA).

### Chemical Analyses

Diet samples were analyzed for dry matter by oven drying at 135°C for 2 h (method 930.15; AOAC Int., 2007) and for ash (method 942.05; AOAC Int., 2007). The gross energy in diets was measured using an isoperibol bomb calorimeter (model 6400, Parr Instruments, Moline, IL, USA) with benzoic acid used as the standard for calibration. Total starch was analyzed by the glucoamylase procedure (method 979.10; AOAC Int., 2007), which yields the enzymatically hydrolyzed starch in the sample. Diets were analyzed for N (method 990.03; AOAC Int., 2007) using a Leco Nitrogen Determinator (model FP628, Leco Corp., St. Joseph, MI, USA) and crude protein was calculated as N × 6.25. Diets were analyzed for insoluble dietary fiber and soluble dietary fiber on an Ankom Total Dietary Fiber Analyzer (Ankom Technology, Macedon, NY, USA) using method 991.43 (AOAC Int., 2007). Total dietary fiber was calculated as the sum of analyzed insoluble and analyzed soluble dietary fiber. Acid-hydrolyzed ether extract in diets was measured by crude fat extraction using petroleum ether (Ankom^XT15^, Ankom Technology, Macedon, NY, USA) following hydrolysis using 3*N* HCl (Ankom^HCl^, Ankom Technology, Macedon, NY, USA). Amino acids were analyzed at the University of Missouri Agricultural Experiment Station (Columbus, MO). Diets were analyzed for amino acids on a Hitachi Amino Acid Analyzer, Model No. L8800 (Hitachi High Technologies America, Inc; Pleasanton, CA, USA) using ninhydrin for post-column derivatization and norleucine as the internal standard. Prior to analysis, samples were hydrolyzed with 6*N* HCl for 24 h at 110°C [method 982.30 E(a); AOAC Int., 2007]. Methionine and Cys were determined as Met sulfone and cysteic acid after cold performic acid oxidation overnight before hydrolysis [method 982.30 E(b); AOAC Int., 2007]. Tryptophan was determined after NaOH hydrolysis for 22 h at 110°C [method 982.30 E(c); AOAC Int., 2007]. The corn and hybrid rye used in the experiment were analyzed for mycotoxins at Trilogy Analytical Laboratories (Washington, MO, USA) using liquid chromatography-tandem mass spectroscopy. The detection limit was 0.1 mg/kg for 15-acetyl deoxynivalenol, 3-acetyl deoxynivalenol, deoxynivalenol, fumonisin (B1, B2, and B3), fusarenon X, and nivalenol. Citrinin and diacetoxyscirpenol had a detection limit of 0.05 mg/kg, neosolaniol had a detection limit of 0.02 mg/kg. The detection limit for zearalenone was 0.0125 mg/kg, the detection limits for HT-2 and T-2 toxins were 0.005 mg/kg, and detection limits for aflatoxin B1, B2, G1, and G2 and for ochratoxin A was 0.001 mg/kg. Analysis of ergot alkaloids in hybrid rye was conducted by refractive index high-performance liquid chromatography using Phenomenex Strata-X-CW weak cation exchange and a reversed-phase column with a detection limit of 10 µg/kg (Phenomenex, Inc., Torrance, CA, USA).

### Calculations and Statistical Analyses

Pen was the experimental unit and data were summarized for each treatment group. Average daily gain (ADG), ADFI, gain:feed (G:F), average fecal scores, and diarrhea frequency were calculated for each of the three phases as well as for the entire experiment. Data were analyzed using SAS 9.4 (SAS Institute Inc., Cary, NC, USA). The normality of the residuals was confirmed, and outliers were tested for using the UNIVARIATE procedure of SAS. Outliers were defined as observations with internally studentized residuals less than −3 or greater than 3, but no outliers were detected. With the exception of diarrhea frequency, data were analyzed by the MIXED procedure, and the statistical model included diet as the fixed effect and replicate as the random effect. The least-square means were calculated for each treatment group using the LSMEANS statement in PROC MIXED. Contrast statements were used to test for linear and quadratic effects of using increasing concentrations of hybrid rye in the diets. Diarrhea frequency was analyzed by the GLIMMIX procedure with a binomial distribution and was expressed as the proportion of the number of observations of fecal scores 3 or more divided by the total number of observations. Results were considered significant at *P* ≤ 0.05 and considered a trend at 0.05 < *P* ≤ 0.10.

## RESULTS

The chemical compositions of diets were within the expected ranges (Table 2). No mycotoxins were detected in the corn used in phase 1 diets, but 0.2 mg/kg fumonisin B1, 0.2 mg/kg deoxynivalenol, 0.03 mg/kg zearalenone, and 0.01 mg/kg HT-2 toxin were detected in corn fed in phases 2 and 3. Ergot alkaloids were not detected in the hybrid rye used in the experiment, but the hybrid rye contained 10 µg/kg of HT-2 toxin.

**Table 2.** Analyzed composition of diets with increasing replacement of corn with hybrid rye (as-is basis)

	Phase 1^1^				Phase 2^1^				Phase 3^1^			
Item	0%	20%	40%	60%	0%	30%	60%	90%	0%	33%	66%	100%
Dry matter, %	89.04	89.03	88.93	89.30	88.73	88.54	88.54	88.14	87.80	87.63	87.89	87.73
Ash, %	6.07	6.08	6.00	6.37	5.60	5.26	5.31	5.42	4.69	4.48	5.21	5.01
Gross energy, kcal/kg	3,972	4,022	4,039	4,007	4,005	4,020	3,988	3,984	4,016	3,998	3,978	3,962
ME^2^, kcal/kg	3,460	3,441	3,421	3,402	3,409	3,371	3,333	3,295	3,367	3,319	3,272	3,223
Starch, %	20.45	20.67	19.79	19.80	28.85	29.66	26.18	25.25	34.80	31.20	33.09	25.24
Crude protein, %	22.88	22.94	23.66	23.48	21.67	22.07	21.61	22.18	20.90	21.97	22.06	23.00
IDF^3^, %	8.90	8.40	9.10	9.20	9.40	10.20	11.50	11.70	11.40	13.70	13.90	14.60
SDF^3^, %	0.90	1.40	2.10	2.80	0.80	2.10	1.70	2.60	1.10	1.40	1.60	3.10
TDF^3^, %	9.80	9.80	11.20	12.00	10.20	12.20	13.20	14.30	12.50	15.00	15.40	17.70
AEE^3^, %	4.46	4.36	4.11	4.04	4.68	4.51	4.22	4.21	4.67	4.00	3.81	2.76
Amino acids, %												
Arg	1.26	1.40	1.32	1.34	1.26	1.27	1.19	1.29	1.33	1.31	1.32	1.43
His	0.54	0.59	0.56	0.57	0.52	0.53	0.50	0.52	0.53	0.52	0.52	0.56
Ile	1.00	1.09	1.04	1.06	0.96	0.95	0.90	0.95	0.92	0.92	0.92	1.02
Leu	1.87	2.03	1.88	1.87	1.74	1.67	1.54	1.58	1.70	1.64	1.56	1.65
Lys	1.59	1.74	1.66	1.68	1.53	1.53	1.45	1.50	1.36	1.32	1.31	1.42
Met	0.45	0.50	0.45	0.48	0.44	0.45	0.43	0.47	0.36	0.36	0.41	0.41
Cys	0.38	0.41	0.39	0.41	0.31	0.32	0.31	0.34	0.32	0.31	0.32	0.35
Phe	1.03	1.16	1.08	1.08	0.99	0.98	0.95	1.01	1.04	1.04	1.03	1.13
Thr	0.97	1.07	1.00	1.02	0.89	0.86	0.83	0.91	0.79	0.79	0.78	0.89
Trp	0.30	0.30	0.28	0.28	0.25	0.25	0.28	0.26	0.26	0.27	0.27	0.29
Val	1.16	1.30	1.22	1.23	1.02	1.03	0.98	1.04	0.98	1.00	1.00	1.11

^1^The percentages indicate the amount of corn that was replaced with hybrid rye in the diets.

^2^ME = metabolizable energy. Calculated concentration of ME based on the value 3,150 kcal/kg ME in hybrid rye (McGhee and Stein, 2020), and values for all other ingredients were obtained from NRC (2012).

^3^IDF = insoluble dietary fiber; SDF = soluble dietary fiber; TDF = total dietary fiber; AEE = acid-hydrolyzed ether extract.

The body weight of pigs at the end of phase 1, phase 2, and phase 3 was not influenced by dietary treatment (Table 3). ADG in phase 1 linearly increased (*P* < 0.05) as the inclusion of hybrid rye increased, but ADG in phase 2 and phase 3 was not impacted by treatment. ADFI linearly increased (*P* < 0.05) during phase 1, and overall as hybrid rye was added to the diets. The G:F also increased (linear, *P* < 0.05) in phase 1 as the concentration of hybrid rye in the diet increased, but in phase 2, phase 3, and for the overall period, G:F decreased at the greatest inclusion level of hybrid rye in the diets (quadratic, *P* < 0.05). No differences in average fecal scores or in diarrhea incidence were observed (Table 4).

**Table 3.** Growth performance of pigs fed diets containing increasing concentrations of hybrid rye in a three-phase feeding program during the initial 35 d post-weaning^1^

	Corn replacement rate, %^2^					*P*-values	
Item	0/0/0	20/30/33	40/60/66	60/90/100	SEM	Linear	Quadratic
Average body weight, kg							
Day 1	5.63	5.63	5.62	5.63	0.169	0.996	0.968
Day 7	5.86	6.17	6.09	6.26	0.180	0.179	0.706
Day 21	8.76	8.91	9.07	8.95	0.331	0.625	0.684
Day 35	16.73	16.46	16.93	16.07	0.520	0.521	0.566
Average daily gain, kg							
Phase 1, days 1 to 7	0.033	0.078	0.067	0.090	0.011	0.004	0.349
Phase 2, days 8 to 21	0.207	0.196	0.214	0.192	0.015	0.704	0.719
Phase 3, days 22 to 35	0.603	0.581	0.605	0.550	0.020	0.155	0.438
Overall, days 1 to 35	0.327	0.319	0.333	0.310	0.013	0.523	0.553
Average daily feed intake, kg							
Phase 1, days 1 to 7	0.085	0.119	0.115	0.128	0.009	0.004	0.256
Phase 2, days 8 to 21	0.314	0.295	0.317	0.347	0.020	0.193	0.240
Phase 3, days 22 to 35	0.814	0.777	0.828	0.925	0.042	0.050	0.121
Overall, days 1 to 35	0.487	0.466	0.496	0.551	0.024	0.047	0.120
Gain:feed							
Phase 1, days 1 to 7	0.277	0.650	0.566	0.703	0.086	0.004	0.179
Phase 2, days 8 to 21	0.669	0.661	0.666	0.559	0.024	0.006	0.042
Phase 3, days 22 to 35	0.748	0.747	0.734	0.607	0.023	<0.001	0.010
Overall, days 1 to 35	0.675	0.683	0.672	0.569	0.026	<0.001	0.002

^1^Least-square means for dietary treatments represent eight observations.

^2^The percentages indicate the amount of corn that was replaced with hybrid rye in the diets. The first treatment contained 0% hybrid rye in all phases; in the second treatment, hybrid rye replaced 20, 30, and 33% of the corn in phases 1, 2, and 3 diets, respectively; in the third treatment, hybrid rye replaced 40, 60 and 66% of the corn in phases 1, 2, and 3 diets, respectively; and in the fourth treatment, hybrid rye replaced 60, 90 and 100% of the corn in phases 1, 2, and 3 diets, respectively.

**Table 4.** Average fecal scores and incidence of diarrhea in pigs fed diets containing increasing concentrations of hybrid rye in a three-phase feeding regimen during the initial 35 d post-weaning^1^

	Corn replacement rate, %^2^					*P*-values	
Item	0/0/0	20/30/33	40/60/66	60/90/100	SEM	Linear	Quadratic
Average fecal score^3^							
Phase 1, days 1 to 7	1.98	2.19	2.38	2.17	0.136	0.228	0.135
Phase 2, days 8 to 21	2.78	2.72	2.87	2.69	0.101	0.800	0.516
Phase 3, days 22 to 35	1.54	1.64	1.82	1.72	0.135	0.233	0.455
Overall, days 1 to 35	2.13	2.18	2.35	2.20	0.083	0.306	0.207
Diarrhea incidence^4^, %							
Phase 1, days 1 to 7	25.00	33.33	37.50	29.17	9.882	0.693	0.386
Phase 2, days 8 to 21	51.79	55.36	57.14	48.21	6.677	0.768	0.357
Phase 3, days 22 to 35	0.00	10.71	3.57	8.29	4.133	0.971	0.972
Overall, days 1 to 35	25.74	33.09	31.62	28.68	4.035	0.663	0.199

^1^Least-square means for dietary treatments represent eight observations.

^2^The percentages indicate the amount of corn that was replaced with hybrid rye in the diets. The first treatment contained 0% hybrid rye in all phases; in the second treatment, hybrid rye replaced 20, 30, and 33% of the corn in phases 1, 2, and 3 diets, respectively; in the third treatment, hybrid rye replaced 40, 60 and 66% of the corn in phases 1, 2, and 3 diets, respectively; and in the fourth treatment, hybrid rye replaced 60, 90 and 100% of the corn in phases 1, 2, and 3 diets, respectively.

^3^Fecal scores: 1 = normal feces; 2 = moist feces; 3 = mild diarrhea; 4 = severe diarrhea; and 5 = watery diarrhea.

^4^Incidence of diarrhea calculated as number of days with a fecal score 3 or more divided by total number of observation.

On days 21 and 35, blood urea N linearly increased (*P* < 0.05) as hybrid rye was added to the diets; and on day 21, serum total protein also linearly increased (*P* < 0.05) with hybrid rye inclusion (Tables 5 and 6). The concentration of red blood cells and hemoglobin in the blood was not affected by diet, but the mean corpuscular hemoglobin concentration on d 35 increased and then decreased as diet hybrid rye inclusion increased (quadratic, *P* < 0.05). Neutrophils on d 21 tended to increase (linear, *P* < 0.10) with increased hybrid rye inclusion in the diet and a tendency (quadratic, *P* < 0.10) for decreased neutrophils at the intermediate levels and then increased neutrophils at the highest level of hybrid rye on d 35 was also observed. Eosinophils on day 21 tended to increase and then decrease as hybrid rye increased in the diets (*P* < 0.10). Lymphocytes on day 35 also tended to increase and then decrease as hybrid rye in the diet increased (quadratic, *P* < 0.10).

**Table 5.** Complete blood count and serum analyses on day 21 post-weaning of pigs fed diets in which increasing proportions of corn were replaced with hybrid rye^1^

	Corn replacement rate, %^2^					*P*-values	
Item	0	30	60	90	SEM	Linear	Quadratic
Red blood cells, × 10^6^/µL	6.36	6.53	6.26	6.39	0.181	0.819	0.918
Hemoglobin, g/dL	10.20	10.35	10.25	9.85	0.267	0.344	0.312
Packed cell volume, %	33.24	33.71	33.36	32.19	0.848	0.363	0.339
Mean corpuscular volume, fl	52.40	51.90	53.68	50.46	1.552	0.565	0.390
Mean corpuscular hemoglobin, pg	16.11	15.93	16.50	15.46	0.529	0.566	0.429
Mean corpuscular hemoglobin concentration, g/dL	30.70	30.72	30.69	30.61	0.258	0.805	0.866
Platelets, × 10^5^/µL	6.48	7.01	6.22	7.33	0.413	0.349	0.490
Total white blood cells^3^, × 10^3^/µL	21.78	25.71	25.43	24.04	1.800	0.426	0.151
Neutrophils, × 10^3^/µL	10.69	12.17	13.98	13.52	1.172	0.060	0.416
Lymphocytes, × 10^3^/µL	9.69	12.20	9.78	9.37	1.164	0.521	0.218
Monocytes, × 10^3^/µL	0.85	0.65	1.11	0.78	0.115	0.614	0.521
Eosinophils, × 10^3^/µL	0.25	0.57	0.25	0.14	0.107	0.196	0.056
Urea N, mg/dL	7.38	9.50	9.75	10.75	1.037	0.033	0.592
Albumin, g/dL	2.43	2.33	2.46	2.35	0.070	0.783	0.930
Total protein, g/dL	4.20	4.33	4.35	4.50	0.075	0.010	0.869

^1^Least-square means for dietary treatments represent eight observations.

^2^The percentages indicate the amount of corn that was replaced with hybrid rye in phase 2 diets.

^3^Band cells (or immature neutrophils) and basophils were quantified but not detected in most samples. Therefore, the data did not follow a normal distribution and were excluded from the table.

**Table 6.** Complete blood count and serum analyses on day 35 post-weaning of pigs fed diets in which increasing proportions of corn were replaced with hybrid rye^1^

	Corn replacement rate, %^2^					*P*-values	
Item	0	33	66	100	SEM	Linear	Quadratic
Red blood cells, × 10^6^/µL	6.65	6.50	6.54	6.70	0.170	0.790	0.359
Hemoglobin, g/dL	11.95	11.51	12.00	11.63	0.239	0.631	0.897
Packed cell volume, %	38.26	36.17	38.14	37.44	0.797	0.882	0.377
Mean corpuscular volume, fl	57.68	55.79	58.57	55.91	1.275	0.645	0.758
Mean corpuscular hemoglobin, pg	18.03	17.79	18.41	17.36	0.414	0.443	0.320
Mean corpuscular hemoglobin concentration, g/dL	31.24	31.86	31.44	31.08	0.217	0.333	0.027
Platelets, × 10^5^/µL	5.43	5.09	5.94	5.79	0.448	0.317	0.831
Total white blood cells^3^, × 10^3^/µL	12.79	13.40	16.00	15.83	1.618	0.118	0.813
Neutrophils, × 10^3^/µL	4.32	3.53	4.24	6.18	0.810	0.077	0.095
Lymphocytes, × 10^3^/µL	7.36	9.02	11.05	8.69	1.072	0.208	0.068
Monocytes, × 10^3^/µL	0.70	0.60	0.64	1.07	0.213	0.225	0.219
Eosinophils, × 10^3^/µL	0.17	0.20	0.06	0.13	0.051	0.226	0.654
Urea N, mg/dL	10.75	12.75	11.88	13.63	0.731	0.025	0.866
Albumin, g/dL	2.84	2.71	2.86	2.83	0.084	0.767	0.607
Total protein, g/dL	4.76	4.70	4.76	4.89	0.091	0.290	0.311

^1^Least-square means for dietary treatments represent eight observations.

^2^The percentages indicate the amount of corn that was replaced with hybrid rye in phase 3 diets.

^3^Band cells (or immature neutrophils) and basophils were quantified but not detected in most samples. Therefore, the data did not follow a normal distribution and were excluded from the table.

In serum from pigs on day 21, the concentration of IL-1β linearly increased (*P* < 0.05) and IL-1RA tended to increase (linear, *P* < 0.10) with greater inclusion of hybrid rye in the diet (Table 7). The concentration of IL-2 and IL-10 decreased and then increased on day 21 as hybrid rye inclusion in the diet increased (quadratic, *P* < 0.05). On day 35, the concentration of IL-1RA tended to increase and then decrease with higher inclusion levels of hybrid rye in the diet (quadratic, *P* < 0.10), and IL-8, and IL-12 increased and then decreased (quadratic, *P* < 0.05) as hybrid rye in diets increased (Table 8). The concentration of IFN-γ on day 35 decreased and then increased (quadratic, *P* < 0.05) as hybrid rye in diets increased.

**Table 7.** Serum analyses for cytokines and inflammatory markers on day 21 post-weaning of pigs fed diets in which increasing proportions of corn were replaced with hybrid rye^1^

	Corn replacement rate, %^2^					*P*-values	
Item	0	30	60	90	SEM	Linear	Quadratic
IL^3^-1α	7.8	4.8	8.2	16.6	5.26	0.267	0.310
IL-1β	68.6	45.1	86.7	143.0	26.42	0.047	0.146
IL-1RA^3^	266.5	293.8	272.0	407.4	48.21	0.097	0.333
IL-2	134.5	47.0	77.8	148.5	41.98	0.664	0.050
IL-4	166.5	74.4	92.4	225.9	71.27	0.624	0.107
IL-6	24.1	9.5	17.0	28.3	10.89	0.672	0.204
IL-8	254.0	326.6	487.0	378.7	84.67	0.138	0.292
IL-10	318.0	137.7	161.0	287.7	63.88	0.910	0.018
IL-12	818.7	929.0	991.1	991.1	87.34	0.139	0.506
IL-18	674.4	548.3	600.8	855.8	111.09	0.286	0.102
IFN-γ^3^	1,775.6	1,474.0	1,422.7	1,168.0	622.82	0.512	0.990
TNF-α^3^	43.0	25.6	49.0	56.4	10.47	0.172	0.182
IgG^3^, mg/mL	3.12	3.90	3.77	5.09	0.831	0.132	0.749

^1^Least-square means for dietary treatments represent eight observations.

^2^The percentages indicate the amount of corn that was replaced with hybrid rye in phase 2 diets.

^3^IL = interleukin; IL-1RA = IL-1 receptor antagonist; IFN-γ = interferon-gamma; TNF-α = tumor necrosis factor-α; IgG = Immunoglobulin G.

**Table 8.** Serum analyses for cytokines and inflammatory markers on day 35 post-weaning of pigs fed diets in which increasing proportions of corn were replaced with hybrid rye^1^

	Corn replacement rate, %^2^					*P*-values	
Item	0	33	66	100	SEM	Linear	Quadratic
IL^3^-1α	4.1	10.5	9.0	11.5	6.06	0.263	0.560
IL-1β	52.0	86.7	62.5	71.0	31.66	0.709	0.605
IL-1RA^3^	295.9	544.1	278.6	238.8	65.03	0.137	0.056
IL-2	53.9	73.1	36.2	67.1	36.58	0.985	0.769
IL-4	86.3	134.6	70.6	88.1	71.66	0.823	0.849
IL-6	13.8	33.4	21.9	40.3	16.74	0.183	0.774
IL-8	278.1	657.2	632.7	490.7	94.91	0.054	0.006
IL-10	212.8	226.4	131.7	165.7	76.06	0.395	0.807
IL-12	977.2	1,082.9	1,095.8	797.3	93.12	0.161	0.034
IL-18	504.9	609.5	473.3	562.3	132.27	0.934	0.968
IFN-γ^3^	2,323.3	226.0	635.8	1,075.6	580.65	0.457	0.002
TNF-α^3^	51.8	35.4	47.8	33.7	16.77	0.441	0.956
IgG^3^, mg/mL	1.82	1.74	2.64	2.23	0.470	0.317	0.735

^1^Least-square means for dietary treatments represent eight observations.

^2^The percentages indicate the amount of corn that was replaced with hybrid rye in phase 3 diets.

^3^IL = interleukin; IL-1RA = IL-1 receptor antagonist; IFN-γ = interferon-gamma; TNF-α = tumor necrosis factor-α; IgG = Immunoglobulin G.

## DISCUSSION

Hybrid rye is a cereal grain that contains more dietary fiber and less starch than wheat, but the concentration of dietary fiber in hybrid rye is less than in barley (McGhee and Stein, 2020). In northern Europe and Canada, barley and wheat are common feed ingredients in diets for pigs, and hybrid rye may replace some or all of the wheat and barley in diets for gestating and lactating sows (Sørensen and Krogsdahl, 2017), weanling pigs (Chuppava et al., 2020; Ellner et al., 2020; Wilke et al., 2020), and growing-finishing pigs (Schwarz et al., 2015, 2016; Bussières, 2018; Smit et al., 2019; Wilke et al., 2021). However, in the United States and many other countries, corn is the dominant cereal grain fed to pigs, and the effects of replacing corn with hybrid rye in diets for weanling pigs are not as easily predicted. Hybrid rye, due to reduced concentrations of lipids and starch, has less metabolizable energy than corn when fed to growing pigs (McGhee and Stein, 2020) and although hybrid rye contains more protein than corn, concentrations of standardized ileal digestible amino acids are close to concentrations in corn because prececal amino acid digestibility in hybrid rye is less than in corn (McGhee and Stein, 2018). More undigested protein reaching the hindgut of young pigs may compromise intestinal health (Wellock et al., 2008; Heo et al., 2009; Kil and Stein, 2010; Gloaguen et al., 2014). In contrast, the dietary fiber in hybrid rye is more fermentable by growing pigs than the fiber in corn (McGhee and Stein, 2020), and hindgut fermentation of rye fiber may improve the intestinal health of humans and pigs (Karppinen et al., 2003; Bach Knudsen et al., 2005; Le Gall et al., 2009; Chuppava et al., 2020). However, soluble fiber, which is present in greater amounts in hybrid rye than in corn, may pose challenges to digestion in newly weaned pigs because of the increased viscosity of the digesta in the small intestine (Agyekum and Nyachoti, 2017).

Results of a previous experiment indicated that ADG, ADFI, and G:F were not impacted during the initial 3 wk post-weaning when modest amounts of hybrid rye (up to 12% from days 1 to 7 and up to 21% during the following 14 d) were fed at the expense of corn in diets for weanling pigs (McGhee and Stein, 2021). In phase 3 (days 21 to 34) of the previous experiment, ADFI increased, whereas G:F decreased as hybrid rye inclusion in the diet increased by up to 60% of the diet. Therefore, it was hypothesized in the present experiment that growth performance would not be reduced if greater amounts of hybrid rye were included at the expense of corn in phases 1 and 2 diets for weanling pigs. In agreement with the previous observations, no reduction in ADG was observed as diet concentrations of hybrid rye increased, but G:F was reduced in phases 2 and 3, which is likely a result of the reduced metabolizable energy in hybrid rye compared with corn. Because pigs had not been exposed to hybrid rye before weaning and because weanling pigs prefer to eat diets containing corn over diets containing hybrid rye (McGhee et al., 2021) it is possible that the unfamiliarity with hybrid rye caused pigs to waste more feed from the feeders as hybrid rye inclusion increased. However, because we did not separate feed disappearance into feed waste and feed consumption, we do not have data to reject or confirm this hypothesis. Although a reduction in the incidence of diarrhea was observed in the first phase of the previous experiment, no positive or negative impact of hybrid rye was observed for fecal scores or diarrhea incidence in the present experiment. The lack of differences observed for diarrhea frequency in the present experiment may be due to the inherent differences among nursery barns, weaning groups, and pathogen loads of pigs in different experiments.

The increase in blood urea N on both sampling days as the inclusion of hybrid rye in the diet increased indicates that protein accretion may have been limited due to the reduced energy intake of the pigs as hybrid rye in the diets increased. As a consequence, amino acid catabolism may have increased and utilization of dietary protein may have been less efficient in pigs fed diets containing hybrid rye (Waguespack et al., 2011; Millet et al., 2018). The tendency for elevated neutrophils on both days 21 and 35 in pigs fed greater amounts of hybrid rye may indicate a greater activation of the innate immune response; neutrophils defend against infections and produce inflammatory cytokines (Swanson et al., 2002). Likewise, IL-8, a neutrophil recruiter (Kucharzik et al., 2005), was also elevated on day 35 in pigs fed hybrid rye. However, because neutrophils tended to be reduced among pigs fed the medium levels of hybrid rye on day 35 it is possible that a small amount of hybrid rye in the diet for weanling pigs may reduce inflammation and immune system activation, but after a certain threshold, a trend for greater inflammation emerges, as has been previously reported for weanling pigs (Wilke et al., 2020).

Microbial fermentation of rye fiber increases the quantity and proportion of butyrate in the hindgut digesta, and butyrate exerts anti-inflammatory effects in the host (Hamer et al., 2008); however, most serum indicators of inflammation in the present experiment indicated the opposite was true for pigs fed greater amounts of hybrid rye. Indeed, pro-inflammatory cytokines IL-1β, IL-8, and IL-12 were greater in pigs fed greater amounts of hybrid rye, whereas anti-inflammatory cytokines IL-2 and IL-10 were reduced on at least one sampling day (day 21) at the lower or intermediate inclusion level of hybrid rye. If very large inclusions of hybrid rye are fed to young pigs with immature gastrointestinal tracts, it is possible the dietary fiber and undigested amino acids aggravate, rather than preserve, intestinal epithelium barrier function, thus promoting a pro-inflammatory response and immune system activation. Pro-inflammatory stress in livestock production is associated with economic loss because nutrient and energy utilization shifts away from body mass accretion and toward immune system function (Elsasser et al., 2008). However, the lack of differences in growth rate and fecal score despite the observed differences in immune parameters as hybrid rye in diets increased indicates that the changes in immune cell and cytokine concentrations may not be economically important. More research is warranted to determine the effects of feeding large quantities of hybrid rye to newly weaned pigs on intestinal morphology and immune function, especially under challenged conditions.

## CONCLUSIONS

It was hypothesized that hybrid rye may replace a majority of corn in diets for pigs without jeopardizing growth performance during the initial 5 wk post-weaning. Therefore, hybrid rye was included in nursery diets at inclusion rates up to 24, 48, and 60% in phase 1 (week 1), phase 2 (weeks 2 and 3), and phase 3 (weeks 4 and 5). Average body weights and ADG were not impacted by replacing up to 60, 90, and 100% of corn in diets fed to pigs in phases 1, 2, and 3, respectively. However, as hybrid rye inclusion in the diet increased, ADFI increased and G:F was reduced, which was likely a result of the reduced concentration of metabolizable energy in hybrid rye compared with corn. Differences in pro- and anti-inflammatory cytokine concentrations indicate pigs fed hybrid rye had changed immune responses compared with pigs fed corn, but these differences did not influence growth or incidence of diarrhea under the conditions of this experiment.
